# Bee-Parasitic Strepsipterans (Strepsiptera: Stylopidae) Induce Their Hosts’ Flower-Visiting Behavior Change

**DOI:** 10.1093/jisesa/ieab066

**Published:** 2021-09-03

**Authors:** Yuta Nakase, Makoto Kato

**Affiliations:** 1 Department of Biology, Faculty of Science, Shinshu University, Nagano, Japan; 2 Graduate School of Human and Environmental Studies, Kyoto University, Kyoto, Japan

**Keywords:** strepsiptera, stylopization, host–parasite interactions, pollinator parasite, manipulation

## Abstract

Parasites sometimes manipulate their host’s behavior to increase their own fitness by enhancing the likelihood that their offspring will reach their hosts. Bees are often parasitized by immobile adult female strepsipterans which seem to modify bees’ behavior to facilitate the release of mobile first-instar larvae onto flowers. To better understand how the parasite may modify the host’s behavior, we compared the foraging behavior of the sweat bee *Lasioglossum apristum* (Vachal, 1903) (Hymenoptera: Halictidae) between bees parasitized and unparasitized by the strepsipteran *Halictoxenos borealis* Kifune, 1982 (Strepsiptera: Stylopidae). Both parasitized and unparasitized bees frequently visited *Hydrangea serrata* (Thunb.) (Cornales: Hydrangeaceae) inflorescences, which are polleniferous but nectarless. On *H. serrata* inflorescences, unparasitized bees collected pollen from the anthers, but parasitized bees did not collect or eat pollen. Instead, they displayed a peculiar behavior, bending their abdomens downward and pressing them against the flower. This peculiar behavior, which was observed only in bees parasitized by a female strepsipteran in the larvae-releasing stage, may promote the release of mobile first-instar larvae onto flowers. Our observations suggest that the altered flower-visiting behavior of parasitized bees may benefit the parasite. Moreover, it suggests that strepsipteran parasites may modify their host’s behavior only when the larvae reach a certain life stage.

Many parasites modify the behavior of their hosts in ways that seem to improve the parasites’ survival and increase their fitness (Combes 2001, Moore 2002, Thomas et al. 2005, Poulin 2010, Poulin and Maure 2015, Hughes and Libersat 2019). These changes in host behavior enhance the host-to-host transmission of parasites or parasite offspring by ensuring that the parasite, its larvae, or its propagules are released in an appropriate location, or by increasing the likelihood of the host’s (and thus eventually the parasite’s) survival (Hughes and Libersat 2019).

Strepsiptera is an order of parasitic insects (excepting basal parasitoid groups) that exhibit extreme sexual dimorphism: Strepsipteran adult males are free-living, winged insects that spend their lives seeking females to fertilize (Pohl and Beutel 2005, Kathirithamby 2018). The first-instar larvae are also free living until they find and enter a host (see Fig. 1 for the life cycle of Stylopidae). Females (excluding one basal family; Kathirithamby 2018) are neotenic and completely endoparasitic within their hosts. First-instar larvae of Stylopidae emerge from a female onto a flower and wait for a bee to visit the flower to collect pollen and nectar, and then they attach themselves to the host bee and are carried to its nest.

**Fig. 1.**
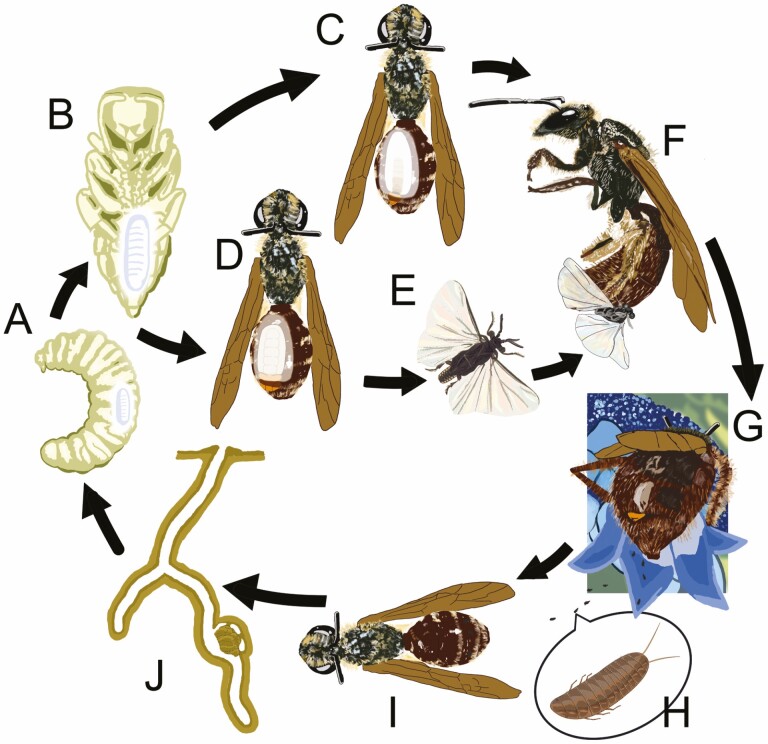
Life history of the strepsipteran *Halictoxenos borealis*. (A and B) The endoparasitic larva grows in the host’s abdomen. (C) The mature female extrudes her cephalothorax in the interstice between tergites IV and V. (D) The maturing male pupates within the host’s abdomen. (E) The adult male emerges from the host. (F) The winged free-living male copulates with an endoparasitic adult female. (G and H) First-instar larvae are released onto a flower. (I and J) First-instar larvae attach themselves to a bee and are carried to its nest, where they parasitize the bee’s offspring.

Host individuals parasitized by Strepsiptera undergo various changes, including loss of fecundity and social behavior (Linsley and MacSwain 1957, Sakagami and Hayashida 1961, Kathirithamby 2018). Although parasitism by Strepsiptera affects the behavior of their hymenopteran hosts (Linsley and MacSwain 1957, Beani et al. 2018, 2020), no studies have distinguished behaviors manipulated to benefit the parasite from other behaviors that benefit the host. Even though parasitized host insects exhibit unusual behaviors, it is difficult to distinguish manipulations that are beneficial to the parasite from behaviors caused by the physical changes induced by the parasite. We distinguish between the two by confirming the flower-visiting behavior of parasitized bees without feeding.

Bees visit flowers to forage and collect nectar and pollen. Generally, bees gather nectar into stomachs and collect pollen in specialized pollen-carrying structures (scopae or corbiculae) on their lower abdomen or their hind legs (Michener 2000). Usually, bees use pollen as food for their larvae and nectar as nutritious resources for their larvae and themselves. Parasitized bees, however, lose their fecundity and no longer exhibit pollen-collecting behavior (Linsley and MacSwain 1957); therefore, the amount of pollen needed for feeding and pollen collection is expected to differ between parasitized and unparasitized bees.

Here we describe the flower-visiting behavior of parasitized bees without feeding. We studied the flower-visiting behavior of bees to plants with flowers that produce no nectar but attract bees by offering a pollen reward. Specifically, we examined the preferences of parasitized and unparasitized individuals of *Lasioglossum apristum* (Hymenoptera: Halictidae) for two sympatric plant species that bloom simultaneously: *Hydrangea serrata* (Cornales: Hydrangeaceae) flowers, which have pollen but no nectar, and *Aruncus dioicus* (Walter) (Rosales: Rosaceae) flowers, which have both nectar and pollen. The strepsipteran parasite *Halictoxenos borealis* (Strepsiptera: Stylopidae) is host-specific to the halictine bee *L. apristum* of Japan (Kifune 1982). In this study, the parasitized bees did not change their preference for flowers, but they did exhibit the behavior of releasing first-instar larvae on the flowers. These suggest that the flower-visiting behavior of parasitized bees is modified by strepsipteran parasites.

## Materials and Methods

**Fig. 2.**
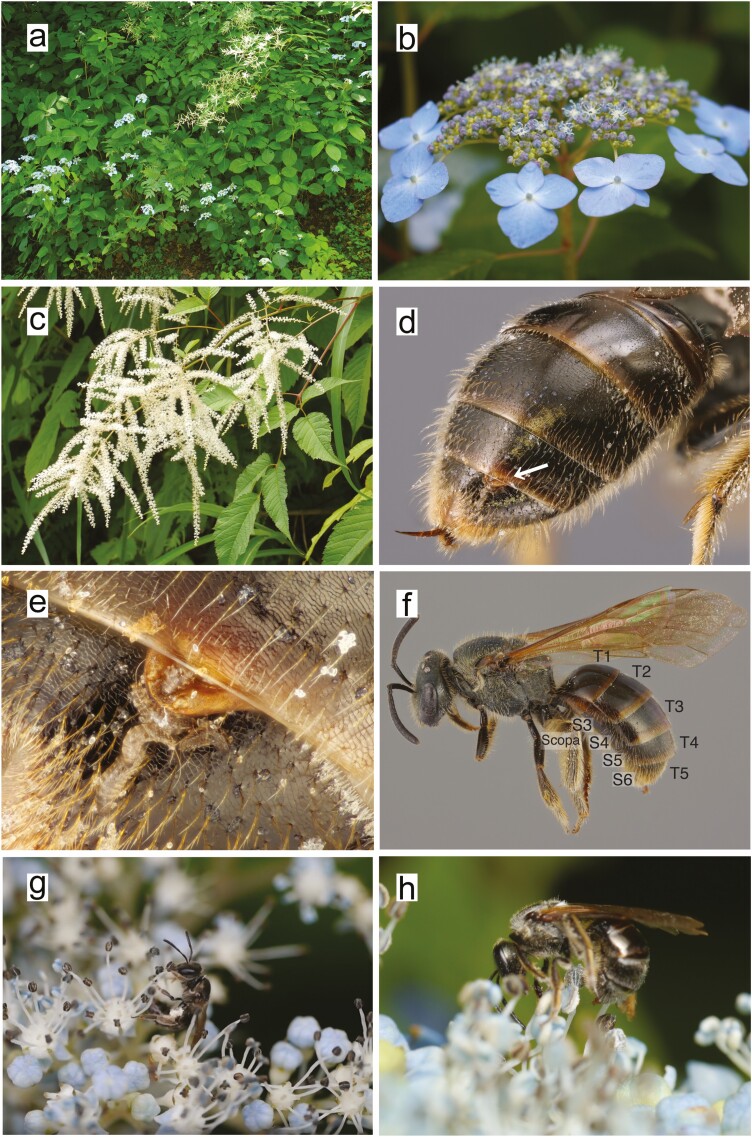
(a) The study site on Mt. Amakazari, Nagano, Japan. *Hydrangea serrata* (blue flowers) and *Aruncus dioicus* (whitish flowers) are sympatrically distributed and bloom simultaneously. (b) A *H. serrata* inflorescence. (c) A *A. dioicus* inflorescence. (d) The cephalothorax of a strepsipteran female (arrow) extruding from the interstice between T4 and T5 on the right side of the abdomen of a female *Lasioglossum apristum*. (e) First-instar larvae emerging from the female cephalothorax via the brood canal opening. (f) Anatomy of *L. apristum*: S3–S6, sternites III to VI; T1–T5, tergites I to V. (g) An unparasitized bee on a *H. serrata* inflorescence collects pollen onto its scopa. (h) A parasitized bee pressing its abdomen against a *H. serrata* inflorescence.

Field surveys of bees were conducted during 17 to 26 July 2010, 16 to 24 July 2011, and 19 to 24 July 2012 on Mt. Amakazari, Otari, Azumino County, Nagano Prefecture, Japan (altitude 1100 m; 36°87′N, 137°98′E). The study site is in a natural deciduous forest dominated by *Fagus crenata*, and the perennial shrubs *Hydrangea serrata* and *Aruncus dioicus*, both of which bloom in July, grow on the forest floor and along forest edges (Fig. 2a). The inflorescence of *H. serrata* is a compound umbel composed of many small, blue hermaphroditic flowers and several blue, sterile decorative flowers; neither type of flower secretes nectar (Fig. 2b). The inflorescence of *A. dioicus* is a panicle composed of small, white nectariferous hermaphroditic flowers (Fig. 2c). During our field surveys, flowers of both species were visited by numerous halictid bees, some of which were parasitized by strepsipterans. Visiting bees were sampled from flowers of each species between 0700 and 1500 h each day from 17 to 26 July 2010 and from 16 to 24 July 2011. All of the collected bees were brought back to the laboratory for species identification and to be examined for sex, parasitism, and pollen attachment. The behaviors recorded (by video) and flowers collected for first-instar larvae detection were conducted during 19 to 24 July 2012. The behavior on the flower was classified into four units, and the time spent on each behavioral unit was measured for all the videos taken in which the start and end of each behavioral unit could be recognized. The four units were 1) walking on flowers, 2) touching antennae and mouth to the flower, 3) collecting pollen, and 4) bending the abdomen downward and pressing the dorsal abdomen against the flower. For more details on the methods, see Supp Data (online only).

## Results

### Parasitism by Strepsipterans

Among the bees collected on *H. serrata* and *Aruncus dioicus* flowers, *Lasioglossum apristum* was the most collected parasitized species (12.3%, 117 of 953 *L. apristum* individuals were parasitized, all by *Halictoxenos borealis*). The number of species and individuals of all bees collected in the 2010 and 2011 sampling, as well as their parasitic strepsipterans, are shown in Supp Table S1 (online only). All collected *L. apristum* bees and their *H. borealis* parasites were female. The results presented below are those for *L. apristum* bees (hereafter, bees) and *H. borealis* parasites (hereafter, strepsipterans). Most parasitized bees were parasitized by a single strepsipteran individual, but eight bees were parasitized by two strepsipterans, and one bee was parasitized by three strepsipterans. None of the parasitized bees collected from *H. serrata* had developed ovaries (*N* = 105), and they were likely infertile. All of the strepsipterans were mature females and contained first-instar larvae in their cephalothorax. The cephalothorax of the strepsipteran females extruded from the right or left side of the membranous portion of their host’s dorsal abdomen, between tergites IV and V (Fig. 2d and e). Parasitized and unparasitized bees visited both *H. serrata* and *A. dioicus* flowers, and the ratio of parasitized to unparasitized bees visiting flowers did not differ significantly between the plant species (105 parasitized, 695 unparasitized from *H. serrata* and 12 parasitized, 141 unparasitized from *A. dioicus*, Pearson’s chi-squared test with Yates’ continuity correction, X-squared = 2.8547, df = 1, *P* = 0.091).

**Fig. 3.**
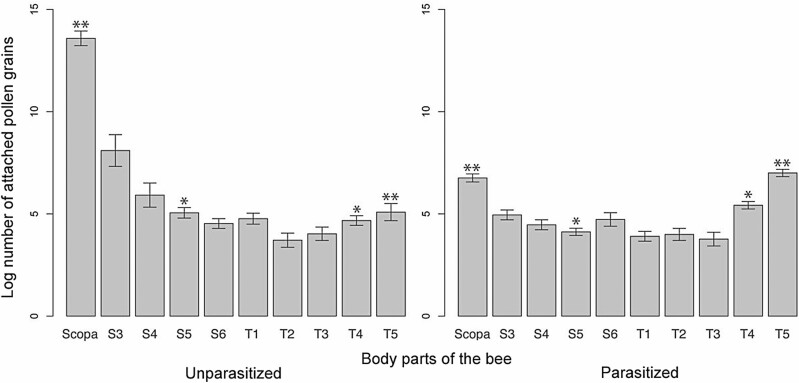
Numbers of pollen grains attached to each body part of unparasitized (left) and parasitized (right) *Lasioglossum apristum* bees. Bars show SE *P < 0.05, ** *P* < 0.0045.

Because both unparasitized and parasitized bees walked around on the inflorescences, their bodies became dusted with pollen, but detailed observations revealed that the amount of pollen on the scopa and abdominal segment T5 differed significantly between unparasitized and parasitized bees (*N* = 22, 11 unparasitized bees and 11 parasitized bees, *P* < 0.0045, Welch two-sample *t*-test with Bonferroni adjustment; Figs. 2f and 3). Pollen attachment on the parasitized bees also exhibit different behavior and do not collect pollen while bending the abdomen downward and pressing the dorsal abdomen against the flower. The gastric contents of parasitized bees consisted of a transparent, yellowish, somewhat sticky liquid that did not contain pollen grains. And the gastric contents of unparasitized bees also did not contain pollen grains.

### Manipulation of Host Bee Behavior by Strepsipteran Parasites

We observed the behavior of parasitized and unparasitized bees on *H. serrata* inflorescences both directly (in the field) and indirectly (on videos). On average, unparasitized bees spent significantly more time on a *H. serrata* inflorescence as compared to parasitized bees (a total of 109 min of recorded video, *N* = 81, 60 unparasitized bees mean time 141 ± 45.4 (SE) s and 21 parasitized bees mean time 32.5 ± 3.0 s; Mann–Whitney *U* test, *W* = 1067, *P* < 0.001). Our observations indicated that unparasitized bees walked on inflorescences, probed the flowers with their antennae and their mouth, and used their forelegs to collect pollen from anthers (Fig. 2g, Supp Video S1 [online only]). In contrast, parasitized bees walked on inflorescences and bent their abdomens downward and pressed them against the flowers without collecting pollen (Fig. 2h, Supp Video S1 [online only]).

**Fig. 4.**
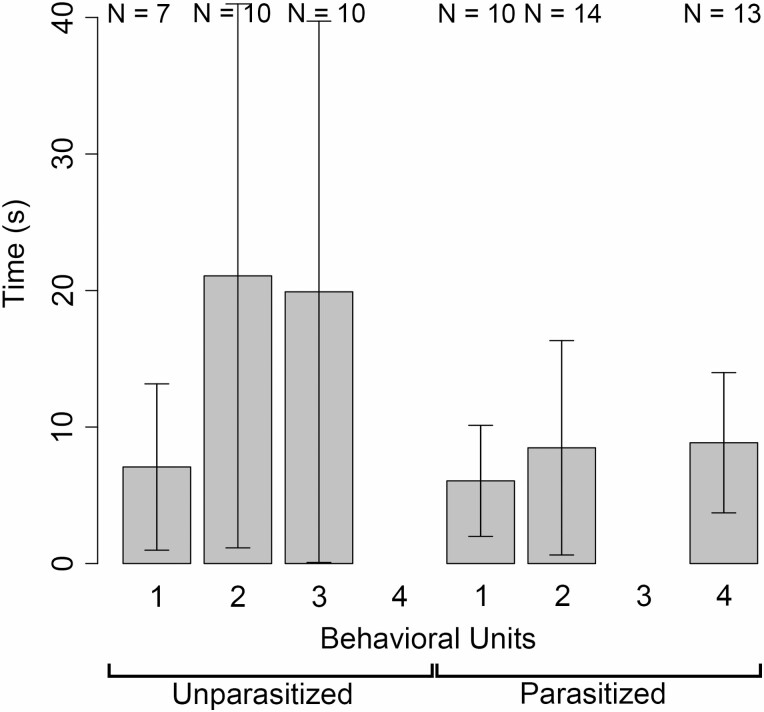
Mean time spent on behavioral units 1–4 by unparasitized and parasitized bees. Bars show SD. The behavioral units 1–4 are respectively as follows: (1) walking on flowers, (2) touching antennae and mouth to the flower, (3) collecting pollen, and (4) bending the abdomen downward and pressing the dorsal abdomen against the flower. Sample size (N) for each behavioral unit is given above each boxplot.

Time sequences of eight bee individuals (three unparasitized and five parasitized; Supp Fig. S1 [online only]) confirmed that bee behavior on flowers differed between unparasitized and parasitized bees. Unparasitized bees exhibited behavioral units in the order 1-2-3, whereas parasitized bees exhibited behavioral units was 1-2-4; to recall, (3) is collecting pollen and (4) is bending the abdomen downward and pressing the dorsal abdomen against the flower. Thus, only unparasitized bees collected pollen, and only parasitized bees exhibited behavioral unit 4. In addition, the mean time spent on behavioral unit 2 (touching antennae and mouth to the flower) was longer in unparasitized bees than parasitized bees (Mann–Whitney *U* test, *W* = 106, *P* = 0.038 < 0.05), whereas the mean time spent on behavioral unit 1 (walking on flower) did not differ significantly between them (Mann–Whitney *U* test, *W* = 40, *P* = 0.669) (Fig. 4). Neither unparasitized nor parasitized bees touched their antennae and mouth to the pistil or its base, where nectar might be secreted. Therefore, even in the unlikely event that nectar was secreted, the bees would not have been feeding on nectar.

### Detection of a Strepsipteran Larva on Flower

On the filters through which the wash solution of the three *H. serrata* inflorescences visited by parasitized bees had been passed, we found one strepsipteran first-instar larva (Supp Fig. S2 [online only]).

## Discussion

In this study, we observed a behavioral change which seems adaptive only for the parasite in the host–parasite relationship. Parasitized bees visited *H. serrata* inflorescences, but did not collect pollen in their scopae, nor have pollen in their gastric contents. Unparasitized bees also did not have pollen in their stomach contents, but they did collect pollen in their scopae (Fig. 3). It is clear that unparasitized bees visited *Hydrangea* flowers to collect pollen, whereas parasitized bees did not visit to forage on pollen or collect nectar as nutritive resources. The parasitized bees showed only behavior to release strepsipteran larvae on the flowers. All strepsipteran parasites on the collected bees were mature females at the stage at which the first-instar larvae are released; no males or immature females parasitized any of the bees collected on flowers. These results suggest that only strepsipteran females at this stage manipulate their host bees to visit flowers (including nectarless flowers such as those of *Hydrangea* species), and the hosts are releasing the larvae of parasites on flowers. There was no significant difference in the proportion of parasitized bees collected on *H. serrata* and *Aruncus dioicus*. We found no evidence that the parasites influence flower preference by their hosts. However, the fact that parasitized bees do not change their preference for flowers may contribute to increasing the efficiency of infection because the first-instar larvae can be released on flowers that are frequently visited by the host species bees. In addition, we found one first-instar larvae on the flowers. This discovery supports the transmission of the first-instar larvae by phoresy via flowers.

The finding that the parasitized bees visited flowers to release the first-instar larvae of their parasites and not to forage for food is evidence that strepsipteran parasites can alter the behavior of individual bees by changing their flower-visiting objective and corresponding behavior from pollen collection to the release of first-instar larvae. Parasitized bees keep visiting the nectarless flowers of *H. serrata*, even though this flower-visiting behavior is not beneficial to host individuals, and it might even have a negative effect on the host, because the host increases both its predation risk and energy consumption while realizing no benefit. However, the behavior of releasing first-instar larvae onto flowers is obviously advantageous for the bees’ strepsipteran parasites. Although the host behavioral changes on *Hydrangea* flowers result from strepsipteran parasitism that is clearly beneficial to the parasite, in order to rigorously test that it is due to manipulation by the parasite, it will be necessary to demonstrate experimentally a physiological control to the changed behavior (Thomas et al. 2005, Bernardo and Singer 2017).

## Supplementary Material

ieab066_suppl_Supplementary_Materials

ieab066_suppl_Supplementary_Video
