# Supplementary material for: Bee-Parasitic Strepsipterans (Strepsiptera: Stylopidae) Induce Their Hosts’ Flower-Visiting Behavior Change
Source: J Insect Sci. 2021 Sep 3;21(5):2. doi: 10.1093/jisesa/ieab066 (PMC8415181; doi:10.1093/jisesa/ieab066)
Supplement: ieab066_suppl_Supplementary_Materials [file ieab066_suppl_supplementary_materials.pdf]

Bee-Parasitic Strepsipterans (Strepsiptera: Stylopidae) Induce Their Hosts' Flower-Visiting Behavior Change

Yuta Nakase[1] and Makoto Kato[2]

[1]Department of Biology, Faculty of Science, Shinshu University, Nagano, Japan

[2]Graduate School of Human and Environmental Studies, Kyoto University, Kyoto, Japan

Corresponding Author:
Yuta Nakase
Department of Biology
Faculty of Science
Shinshu University, Nagano, Japan
Email: yuta.nakase@gmail.com
Ph: 0263-37-3142

Observation of bee behavior

To observe host behaviors affected by parasitism, we recorded the behavior of both parasitized and unparasitized *Lasioglossum apristum* bees at high definition with a video camera (PANASONIC LUMIX G3, H-ES045 lens, recording speed 30 fps) while they were visiting flowers of *Hydrangea serrata*. All the video recorded bees were able to be identified by their appearance as parasitized or not. Then we analyzed the recorded video by comparing time spent on an inflorescence between parasitized and unparasitized bees and by comparing each bee's behavior on the flower. For the latter comparison, we classified the behavior of bees on flowers into the following four units: (1) walking on flowers, (2) touching antennae and mouth to the flower, (3) collecting pollen, and (4) bending the abdomen downward and pressing the dorsal abdomen against the flower. We also observed whether the mouth of the bee touched a pistil or its base, where nectar might be found. Among these four behavioral units, unit 1 is incompatible with the other behavioral units, whereas units 2 and 3 and units 2 and 4 are compatible pairs and often occurred simultaneously. Units 3 and 4 never occur in the same bee. The behavioral repertoires of parasitized and unparasitized bees and the time spent on each behavioral unit were determined from video frames. We measured the mean time spent on each behavioral

unit by each flower-visiting bee and then compared how parasitized and unparasitized bees allocated their flower-visiting time to the behavior units.

Pollen attachment and gastric contents of host bees

Pollen attachment on the body of each of 22 collected *L. apristum* bees was examined. 11 parasitized, 11 unparasitized bees were randomly selected from among the collected bees (695 parasitized and 105 unparasitized). Under a stereomicroscope, we counted the number of pollen grains on each of the following body parts: scopa, sternites III to VI (S3–S6), and tergites I to V (T1–T5) (Fig. 2*f*). All 22 collected *L. apristum* bees were then dissected to examine the developmental stage of their ovaries, their gastric contents, and, in the case of parasitized bees, the developmental stage and sex of their strepsipteran parasites.

Detection of released strepsipteran larvae on flowers

To determine whether there were any first instar strepsipteran larvae on flowers, we sampled three *H. serrata* inflorescences that had been frequently visited by parasitized *L. apristum* bees. Each inflorescence was fixed in 70% ethanol for transport to the laboratory. In the laboratory, the flowers together with 70% ethanol, were washed in a 50 ml plastic container in an ultrasonic cleaner for 10 minutes, after which they were stirred for a while by hand and the washing solution was passed through a paper filter. The paper filter is fine enough that strepsipteran larvae, plant fragments and pollen do not pass through the filter. Then, we examined each filter under a binocular microscope for first instar strepsipteran larvae. This method is prone to missing the first instar larvae. Therefore, it is difficult to discuss quantitatively.

Fig. S1. Allocation of time to each behavioral unit by parasitized (Nos. 1–5) and unparasitized (Nos. 6–8) bees. Behavioral units: (1) "walking on flowers," (2) "touching an anther by mouth and antennae," (3) "pollen collecting," and (4) "bending the abdomen and pressing the dorsal abdomen against the flower."

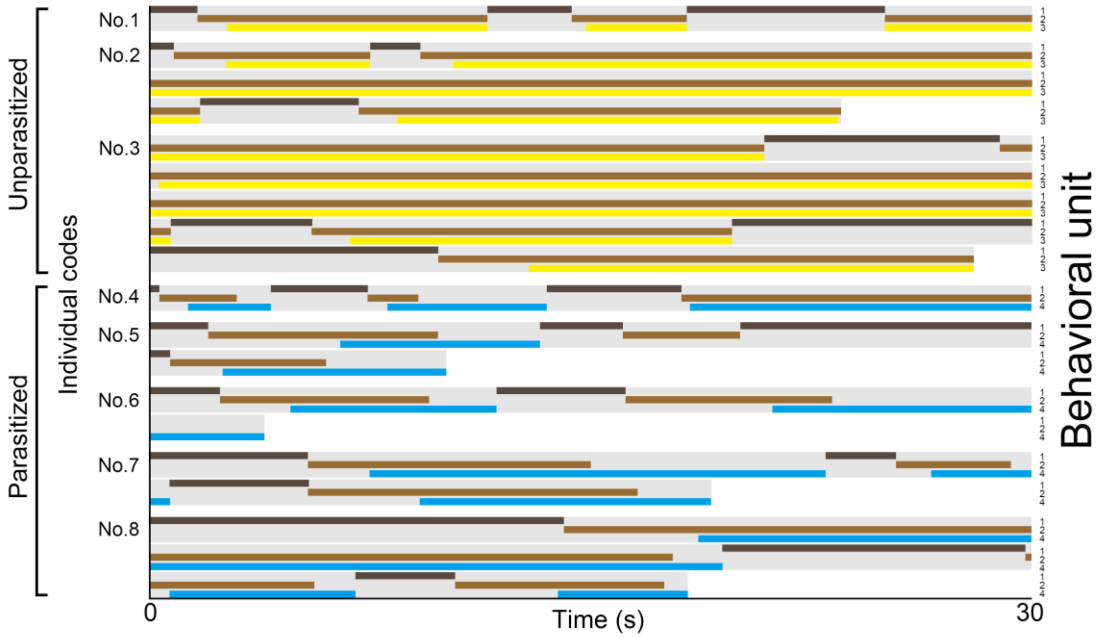

Fig. S2. A first instar strepsipteran larva collected on a *H. serrata* flower. Scale bar: 0.2 mm.

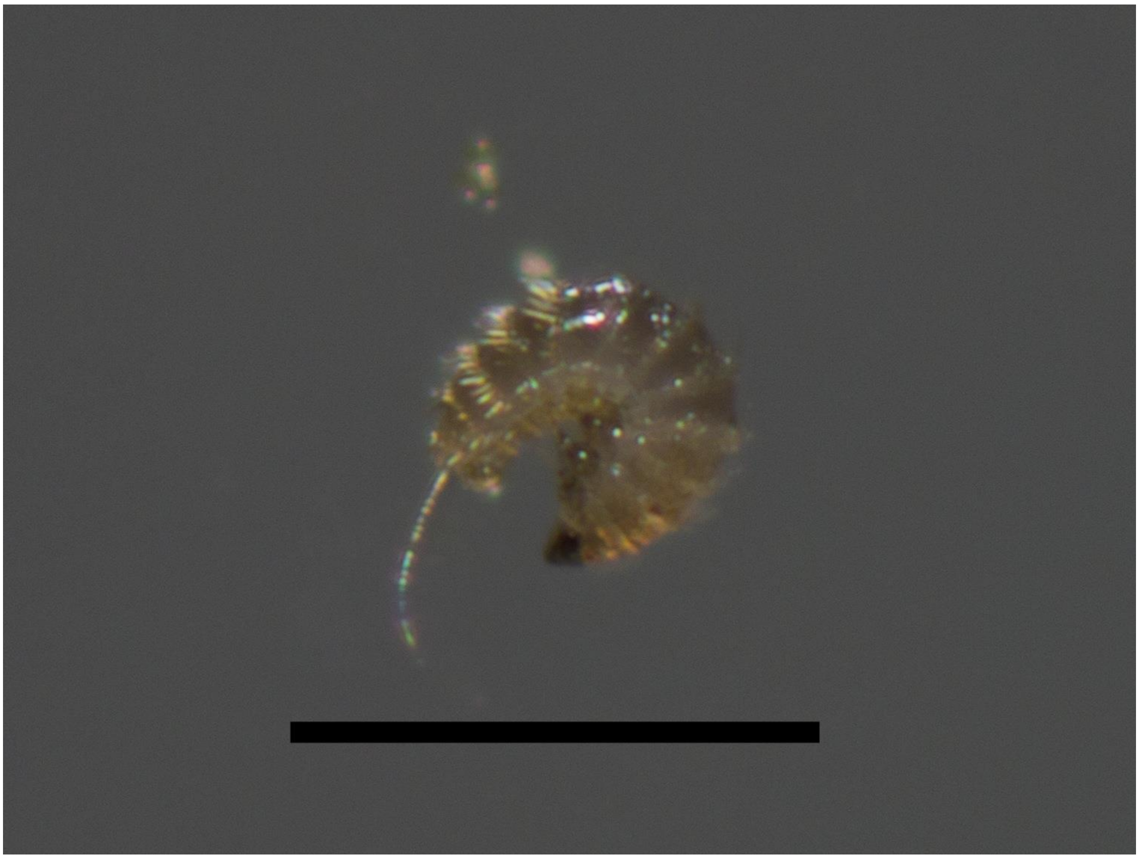

Table S1. Number of individuals of each bee species collected on *H. serrata* and *A. dioicus* flowers. The number of bees that were parasitized is shown in parentheses. Three bee species were parasitized by strepsipterans, *L. apristum*, *L. vulsum*, and *A. subopaca*, which were parasitized by *H. borealis*, *H. latifemoralis*, and *S. kaguyae*, respectively.

Family	Species name	Sociality	*H. serrata*	*A. dioicus*
Halictidae	*Lasioglossum* (*Evylaeus*) *apristum*	Eusocial	800 (105)	153 (12)
	Lasioglossum (*Evylaeus*) *vulsum*	Solitary	139 (12)	36 (6)
	Lasioglossum (*Evylaeus*) *baleicum*	Eusocial	641	107
	Lasioglossum (*Evylaeus*) *sibiriacum*	Eusocial	71	7
	Lasioglossum (*Evylaeus*) *sphecodicolor*	Unknown	65	1
	Lasioglossum (*Evylaeus*) *duplex*	Eusocial	20	4
	Lasioglossum (*Evylaeus*) *transpositum*	Unknown	11	10
	Lasioglossum (*Evylaeus*) *metis*	Unknown	5	1
	Lasioglossum (*Lasioglossum*) *agelastum*	Solitary	28	5
	Lasioglossum (*Lasioglossum*) *kansuense*	Solitary	3	1
	Lasioglossum (*Lasioglossum*) *harmandi*	Solitary	5	5
	Lasioglossum (*Lasioglossum*) *proximatum*	Solitary	21	
	Lasioglossum (*Evylaeus*) *kuroshio*	Unknown	6	
	Lasioglossum (*Evylaeus*) *zunaga*	Unknown		2

	Lasioglossum (*Evylaeus*) spp.	Unknown	76	71
	Specodes spp.	Cleptoparasite		4
Andrenidae	*Andrena* (*Microandrena*) *subopaca*	Solitary	1	171 (14)
	Andrena (*Melandrena*) *parathoracica*	Solitary	10	1
	Andrena (*Hoplandrena*) *akitsushimae*	Solitary	35	
	Andrena (*Microandrena*) *semirugosa brassicae*	Solitary		66
Apidae	*Ceratina* spp.	Solitary	3	48
Colletidae	*Hylaeus* spp.	Solitary	2	83
Crabronidae	*Trypoxylon* sp.	Solitary		4
	Cerceris sp.	Solitary		1
	Alysson japonicus	Solitary		2
	Oxybelus strandi	Solitary		2
Total			1942 (117)	785 (32)
